# Cimicifoetisides A and B, two cytotoxic cycloartane triterpenoid glycosides from the rhizomes of *Cimicifuga foetida*, inhibit proliferation of cancer cells

**DOI:** 10.1186/1860-5397-3-3

**Published:** 2007-01-31

**Authors:** Li-Rong Sun, Chen Qing, Yan-Li Zhang, Shu-Yu Jia, Zhong-Rong Li, Shen-Ji Pei, Ming-Hua Qiu, Michael L Gross, Samuel X Qiu

**Affiliations:** 1 State Key Laboratory of Phytochemistry and Plant Resources in West China, Kunming Institute of Botany, The Chinese Academy of Sciences, Kunming, Yunnan, P. R. China; 2 Yunnan Pharmacological Laboratory of Natural Products, Kunming Medical College, Kunming, Yunnan, P. R. China; 3 Chemistry Department, Washington University, Campus box 1134, One Brookings Drive, St. Louis, MO 63130, USA; 4 Natural Products Drug Discovery, Herbstandard, Inc., 12305 New Avenue, Suite K., Lemont, IL 60439, USA

## Abstract

Two new cycloartane-type triterpene glycosides, namely cimicifoetisides A (**1**) and B (**2**), along with seven known compounds cimigenol, 25-*O*-acetylcimigenol, cimigenol 3-*O*-β-D-xylopyranoside, 12β-hydroxycimigenol 3-*O*-β-D-xylopyranoside, cimigenol 3-*O*-α-L-arabinopyranoside, 25-deoxyshengmanol 3-*O*-β-D-xylopyranoside and cimilactone A, were isolated from the rhizomes of *Cimicifuga foetida*. Their structures were elucidated as cimigenol 3-*O*-(2'-*O*-acetyl)-α-L-arabinopyranoside (**1**) and 25-*O*-acetylcimigenol 3-*O*-(2'-*O*-acetyl)-α-L-arabinopyranoside (**2**). Both compounds **1** and **2** exhibited potent cytotoxicity against rat EAC (Ehrlich ascites carcinoma) and MDA-MB-A231 (human breast cancer) cells with IC_50_ values of 0.52 and 6.74 μM for **1**, and 0.19 and 10.21 μM for **2**, suggesting their potential for further investigation as anti-cancer agents.

## 1. Background

Black cohosh (*Cimicifuga racemosa* (L.) Nutt.), [1] also known as bugbane, is widely used in the United States and the European Union as a herbal dietary supplement for the relief of symptoms related to menopause, [2–3] with a clinical history spanning over the last forty years. [4] Due to the large demand of the plant material to meet the ever-increasing American and European market, the alcoholic extract made from the rhizomes of several species of the same genus native to China, particularly *C. heracleifolia, C. dahurica, C.simplex*, and *C. foetida*, which are used as antipyretic and analgesic agents in traditional Chinese medicines, have been imported into Western markets. [5] In our continuing search for novel anti-cancer agents from natural products, we found that a methanol extract from the rhizomes of *C. foetida* exhibited considerable cytotoxicity to human cancer cell lines. To date, more than 30 triterpene glycosides have been isolated from *C. foetida* collected from different geographic regions. [6–9] In the present investigation on *C. foetida* collected from prefecture of Dali county in Yunnan province, Southern China, two novel glycosides, designated cimicifoetisides A (**1**) and B (**2**), containing a relatively uncommon acetyl-monosaccharide, along with seven known compounds, were isolated and were shown to exhibit potent cytotoxicity against two human cancer cell lines. This report describes the isolation, structure elucidation, and cytotoxicity evaluation of these isolates.

## 2. Results and discussions

**Figure 1:**
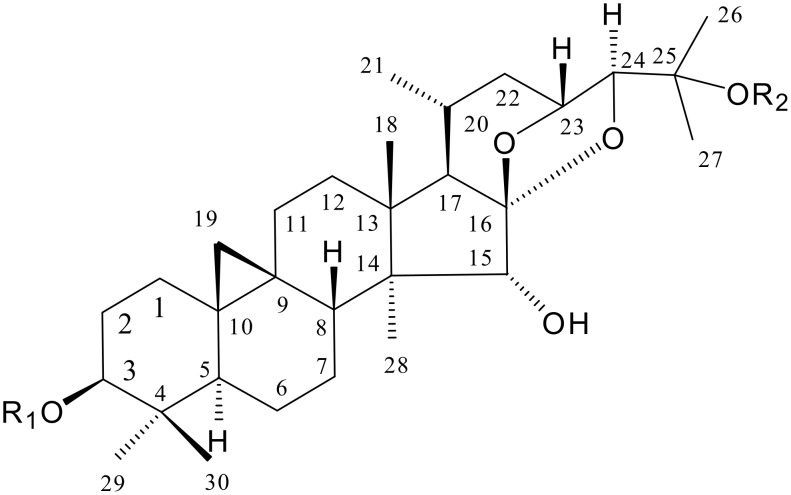
Compounds **1** and **2** isolated from *Cimicifuga foetida*. **1**. R_1_ = 2'-*O*-acetylarabinosyl, R_2_ = H; **2**. R_1_ = 2'-*O*-acetylarabinosyl, R_2_ = Ac

Cimicifoetiside A (**1**) (see Figure 1) exhibited a molecular formula of C_37_H_58_O_10_ based on its ^13^C-NMR DEPT spectrum and negative HRFABMS in which a fragment ion was observed at *m/z* 619.3854 [M-H-OAc]^-^ (calcd for C_35_H_55_O_9_, 619.3846) due to the facile loss of an acetyl group. The overall physical properties and NMR spectral profile revealed its identity as a member of the cycloartane group of triterpene glycosides, a characteristic and distinguishable chemical marker of *Cimicifuga* plants. [6] In the ^1^H-NMR spectrum (Table 1), the characteristic cyclopropane methylene signals at δ_H_ 0.22 and 0.46 (each 1H, d, *J* = 3.0 Hz); eight methyl groups at δ_H_ 0.93, 1.05, 1.12, 1.18, 1.44, 1.47 (each 3H, s), 0.85 (d, *J* = 5.1 Hz), and an acetyl group at δ 2.09 (3H, s); and an anomeric proton at δ_H_ 4.75 (1H, d, *J* = 7.8 Hz) were observed. The ^13^C-NMR and DEPT spectra (Table 1), showed a total of 37 carbon signals, among which, 30 were ascribable to the triterpene aglycone. A characteristic ketalic quaternary carbon signal was observed at δ_C_ 112.0 (s, C-16) together with two oxygen-bearing methine signals at δ_C_ 80.3 (d, C-15) and 90.2 (d, C-24). Two carbons were assigned to an acetyl group [δ_C_ 170.2 and 21.4], and these spectra also showed a set of five oxygenated carbon signals assignable to a pentose moiety [δ_C_ 104.5 (C-1'), 74.4 (C-2'), 72.5 (C-3'), 69.8 (C-4'), and 67.3 (C-5')]. From the above information it was concluded that **1** was a cyclolanostane triterpene linked to a five carbon sugar unit with an acetyl group attached to either the triterpene aglycone or the sugar moiety. But the identities of the triterpene and the monosacchride, the sugar linkage position, and the acetyl group substitution position awaited determination.

**Table 1:** NMR Data for compounds 1 and 2

Position	**1**		**2**			**1**		**2**	
	^1^H	^13^C	^1^H	^13^C		^1^H	^13^C	^1^H	^13^C

1	1.16 m 1.53 m	32.3 t	1.20 m 1.50 m	32.3 t	21	0.85 d 5.1	19.6 q	0.84 d 6.40	19.6 q
2	2.25 m 1.86 dd 2.8, 10.7	30.0 t	2.24 m 1.85 m	30.9 t	22	1.20 m 2.27 m	38.2 t	1.02 m 2.28 m	37.9 t
3	3.44 dd 3.5, 9.3	88.7 d	3.36 dd 4.2, 11.6	88.7 d	23	4.75 d 7.8	71.3 d	4.59 d 9.0	71.7 d
4		41.1 s		41.8 s	24	3.77 s	90.2 d	4.10 s	86.8 d
5	1.28 dd 3.5, 9.3	47.5 d	1.28 dd 3.8, 12.1	47.5 d	25		71.0 s		83.2 s
6	0.70 dd 9.6, 10.2 1.66 m	21.1 t	1.50 m	21.4 t	26	1.44 s	27.2 q	1.67 s	24.0 q
7	1.12 m 1.52 m	26.5 t	1.16 m 2.08 s	26.6 t	27	1.47 s	25.4 q	1.65 s	21.5 q
8	1.64 m	48.7 d	1.67 m	48.7 d	28	1.18 s	11.8 q	1.17 s	11.9 q
9		20.1 s		20.1 s	29	1.05 s	25.4 q	1.07 s	22.3 q
10		26.6 s		26.3 s	30	0.93 s	15.3 q	0.95 s	15.3 q
11	1.02 m 2.07 m	26.3 t	1.17 m 2.11 m	26.5 t	2'-COCH_3_		170.2 s		170.2 s
12	1.56 m 1.67 m	34.2 t	1.53 m 1.63 m	34.0 t	2'-COCH_3_	2.11 s	21.4 q	2.09 s	21.4 q
13		41.9 s		41.8 s	25-COCH_3_				170.1 s
14		47.4 s		47.2 s	25-COCH_3_			1.95 s	22.3 q
15	4.31 s	80.3 d	4.26 s	80.2 d	3-ara				
16		112.0 s		112.5 s	1'	4.73 d 6.7	104.5 d	5.16 d 7.8	104.5 d
17	1.50 m	59.6 d	1.44 d 11.0	59.4 d	2'	5.90 t 6.7	74.4 d	5.89 dd 7.8, 9.2	74.4 d
18	1.12 s	19.6 q	1.13 s	19.6 q	3'	4.17 dd 2.7, 7.6	72.5 d	4.16 dd 3.2, 9.2	72.5 d
19	0.22 d 3.1 0.46 d 2.8	30.8 t	0.22 d 3.8 0.46 d 3.4	30.9 t	4'	4.26 m	69.8 d	4.27 m	69.8 d
20	1.65 m	24.1 d	1.67 m	23.4 d	5'	3.74 m	67.3 t	4.25 m	67.3 t

Mild acidic hydrolysis of **1** afforded an aglycone which was shown to be cimigenol, a non-glycosylated cycloartane triterpene previously isolated from the same source, [9] by direct co-HPTLC comparison with a reference sample we isolated in the current study, indicating that the aglycone structure of **1** is cimigenol. This conclusion is further supported by comparison of the corresponding ^1^H and ^13^C-NMR spectral data of the aglycone portion of **1** with those of cimigenol from the literature, [10] after taking the so-called 'glycosylation effect' [11] into account. Consistently, on glycosylation, a 10.7 ppm downfield shift was observed at C-3 accompanied by up-field shifts for the neighboring carbons C-2 (1.4 ppm) and C-4 (0.1 ppm), thereby indicating the sugar moiety to be attached to the C-3 position of the aglycone cimigenol.

To confirm the structure of the aglycone and the glycosidic connection, and to further elucidate the identity of the sugar moiety, a complete ^1^H and ^13^C-NMR spectral assignment was carried out using a combination of DEPT, COSY, HMQC, and HMBC experiments. The ^1^H-^1^H COSY, combined with the HMQC spectrum revealed that **1** has the following partial structure: -CH_2_CH_2_CH- (corresponding to C_1_ to C_3_); -CHCH_2_CH_2_CH- (due to C_5_-C_8_); -CH_2_CH- (for C_11_ to C_12_); -CHCHCH_2_CHCH- (due to C_17_-C_20_-C_22_-C_23_-C_24_-); a geminal proton pair for CH_2_-19; and a set of signals for a pentose, -CHCHCHCHCH_2_- (C-1' to C-5'). All of these segments were compatible for rings A, B, C, D, and E of a 9, 19-cycloartane-type triterpene linked to a five carbon glycosyl unit.

**Figure 2:**
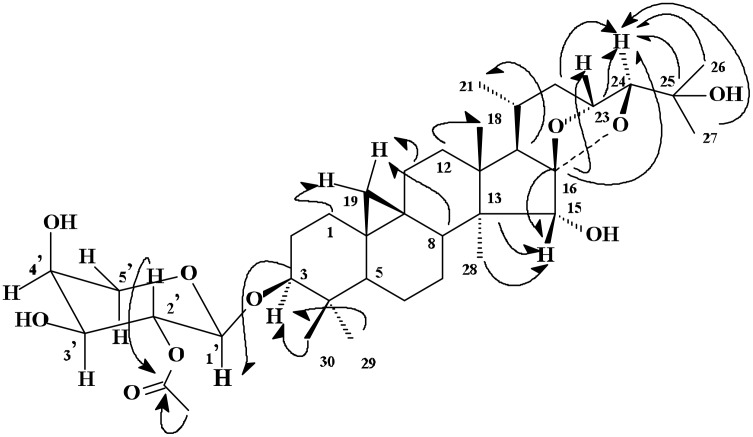
Key long-range ^13^C-^1^H correlations of **1** observed by HMBC.

The HMBC spectrum of **1** provided direct and conclusive evidence for the deduction of the aglycone as cimigenol (See Figure 2). Briefly, significant correlations were observed between the singlet Hβ-15 (4.31) and the signals of two quaternary carbons at δ_C_ 47.4 (C-14) and δ_C_ 112.0 (C-16), and a methyl signal at δ_C_111.8 (C-28); between the H-19 methylene signals (δ_H_ 0.22 and 0.46) and the methylene carbons at δ_C_ 32.3 (C-1) and δ_C_ 26.3 (C-11), a methylene carbon at δ_C_ 48.7 (C-8); between H-3 (δ_H_ 3.44) and methyl carbon signals at δ_C_ 25.4 (C-29) and δ_C_ 15.3 (C-30); between H-23 (δ_H_ 4.75) and the quaternary carbon signal at δ_C_ 112.0 (C-16); between H-24 (δ_H_ 3.77) and the quaternary carbon signals at δ_C_ 112.0 (C-16) and δ_C_ 71.0 (C-25), the methine carbon signal at δ_C_ 71.3 (C-23), the methylene carbon signal at δ_C_ 38.2 (C-22) and the methyl carbon signals at δ_C_ 27.2 (C-26) and 25.4 (C-27). Additionally, the methyl signals at δ_H_ 1.44 (Me-26)/δ_H_ 1.47 (Me-27) showed correlations with a quaternary carbon signal at δ_C_ 71.0 (C-25), the methine carbon signal at δ_C_ 90.2 (C-24), and the methyl carbon signal at δ_C_ 25.4 (C-27)/δ_C_ 27.2 (C-26). Taken together, these data confirmed the aglycone structure of **1** was cimigenol.

In the HMBC spectrum (See Figure 2), an informative correlation was also observed between the anomeric proton signal at δ_H_ 4.73 (H-1', 1H, J = 6.7 Hz) and a methine carbon signal at δ_C_ 88.7 (C-3), implying that the sugar moiety was linked at the C-3 position. The typical large coupling constants between H-1' and H-2' (*J*_H1'-H2'_ = 6.7 Hz), and between H-2' and H-3' (*J*_H2'-H3'_ = 6.7 Hz), as well as the small coupling constant between H-3' and H-4' (*J*_H3'-H4'_ = 2.7 Hz) indicated the sugar moiety is a pentapyranose with the protons at C-1', C-2', and C-3' axially-oriented, while the proton at the C-4' position is equatorially disposed. Thus, the sugar moiety must be either a-*L*-arabinopyranosyl-[^4^C_1_ chair conformation) or β-*D*-arabinopyranosyl [^1^C_4_ chair conformation], with the former being more favorable, as it is a common component of the triterpene glycoside isolated from *Cimicifuga* plants, whereas the isolation of the latter has not been reported. Furthermore, the location of the acetyl group could be unambiguously assigned to C-2' of the arabinose unit by HMBC, as a correlation was observed between H-2' (δ_H_ 5.90, t, *J* = 6.7 Hz) and the carbonyl signal at δ 170.2. On mild alkali hydrolysis with saturated Na_2_CO_3_-MeOH solution, **1** afforded a deacetyl derivative which was shown to be cimigenol 3-α-*L*-arabinopyranoside, which is also a component isolated previously from *CimicifugaI*, [12] and was also isolated in the current investigation, by direct comparison from co-HPTLC and ^1^H-NMR spectroscopy with an authentic sample. Therefore, the structure of cimicifoetiside A (**1**) was unambiguously elucidated as cimigenol 3-*O*-α-*L*-(2'-*O*-acetyl) arabinopyranoside. Further evidence supporting this conclusion was derived by direct comparison of its ^13^C-NMR spectra with those of cimigenol 3-α-*L*-arabinopyranoside. It was found that all of the carbon signals were shown to be superimposable, except for the signals arising from C-1' (3.2 ppm up-field shifted), C-2' (1.2 ppm downfield shifted) and C-3' (2.4 ppm up-field shifted) in **1** compared to cimigenol 3-α-*L*-arabinopyranoside. This could be satisfactorily accounted for by the established 'acylation effect' [13] due to the introduction of an acetyl group at C-2' of cimigenol 3-α-*L*-arabinopyranoside.

**Figure 3:**
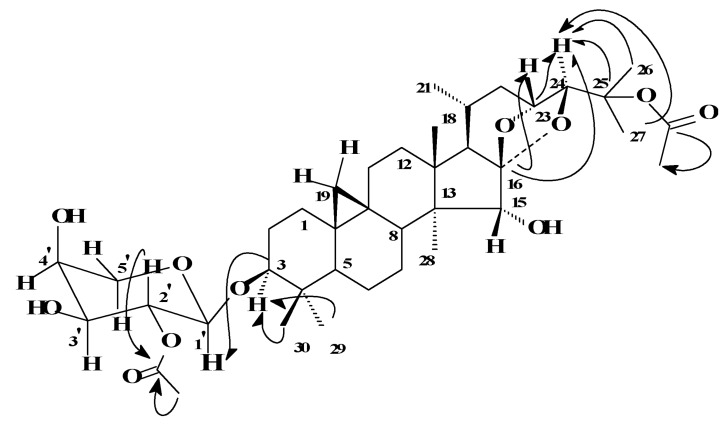
Key long-range ^13^C-^1^H correlations of **2** observed by HMBC for the relevant carbons with the acetyl groups.

Cimicifoetiside B (**2**) was determined to have a molecular formula of C_39_H_60_O_11_ based on its ^13^C-NMR (DEPT) spectral data and HRFABMS, in which a fragment ion was detected at *m/z* 661.3944 [M-H-OAc]^-^ (calcd for C_37_H_57_O_10_, 661.3951) due to the loss of an acetyl group, indicating a 42 a.m.u. increase compared to that of **1**, corresponding to the presence of an additional acetyl group (C_2_H_2_O). This is in agreement with the observation of an extra set of ^1^H and ^13^C-NMR signals for an acetyl group (δ_H_ 1.95, 3H, s; δ_C_ 170.2, s, and 22.3, q). The second acetyl group could be readily attributed to the hydroxyl group at C-25, as a significant downfield shift (12.1 ppm) of C-25, and up-field shifts of C-24 (3.2 ppm), C-26 (3.2 ppm), and C-27 (3.9 ppm) were observed in its ^13^C-NMR spectrum compared with those of **1**, obeying the 'acylation effect' [13] with respect to acetylation occurring at the C-25 position, while the remaining carbon signals were almost identical. Following the same methodology as described for **1**, all of the ^1^H and ^13^C-NMR spectral data of **2** were completely assigned. As shown in Figure 3, the HMBC experiment provided direct and conclusive evidence to assign one acetyl group to the C-2' position of arabinose; while indirect, but compelling, evidence for the assignment of the second acetyl group to C-25 was noted through the unambiguous assignment of the shifted signals of C-24, C-25, C-26, and C-27. The quaternary nature of C-25 prevented the linking of any proton signals to the carbonyl signal from HMBC (See Figure 3), except for the acetyl methyl group. Taken together, the structure of Cimicifoetiside B (**2**) was deduced as 25-*O*-acetylcimigenol 3-*O*-α-*L*-(2'-*O*-acetyl) arabinopyranoside.

The structures of the known compounds were identified as cimigenol, [9] 25-*O*-acetylcimigenol, [14] cimigenol 3-*O*-β-*D*-xylopyranoside, [15] 12β-hydroxycimigenol 3-*O*-β-*D*-xylopyranoside, [16] cimigenol 3-*O*-α-*L*-arabinopyranoside, [12] 25-deoxyshengmanol 3-*O*-β-*D*-xylopyranoside, [15,17] and cimilactone A, [18] by comparing their MS and NMR data to those of the literatures.

It has been reported previously that cimigenol and relative compounds possess cancer chemopreventive activity. [19–20] These results inspire us to explore other activities of this compound family may have. The cytotoxicity of all of the nine compounds was evaluated against the rat tumor EAC cell, and the human SGC7901 and A231 cancer cell lines using an established protocol [21] with a minor modification [see Supporting Information File 1]. As shown in Table 2, both of the two new compounds, especially **2**, exerted significant cytotoxicity against the rat EAC tumor cell line with IC_50_ values of 0.35 and 0.14 μg/ml for **1** and **2**, respectively. Both **1** and **2** also demonstrated moderate inhibition to human MDA-MB-A231 breast cancer cell. While other compounds were shown to be devoid of significant cytotoxicity, implying the higher lipophilic nature of triterpene glycosides could be critical to the cytotoxicity as observed for **1** and **2** whose cytotoxicity are proportional to their lipophilicity (vs. cimigenol 3-*O*-β-*D*-xylopyranoside, which is devoid of appreciable activity) with the introduction of acetyl groups. Interestingly, the aglycones without sugar chains are also devoid of noticeable activity, implying the glycosyl linkage is a prerequisite for optimal cytotoxicity although the SAR and the molecular mechanism underlying the cytotoxicity of these compounds have yet been defined.

**Table 2:** Cytotoxicity of compounds 1 and 2 [IC_50_ μg/mL (μM)]

Test compounds	*EAC*	*SGC7901*	*A231*

**1**	0.35 (0.52)	10~100	4.46 (6.74)
**2**	0.14 (0.19)	10~100	7.19 (10.21)
*cis*-platin*	0.31	0.17	0.87

*positive control; EAC: Ehrlich ascities carcinoma; A231: MDA-MB-A231 breast cancer; SGC7901: human gastric cancer

The results reported herein may suggest the potential for further examination of the cycloartane triterpene glycosides from *Cimicifuga* for the prevention or treatment of human cancers, especially for breast cancer. It should be noted that the extracts of *Cimicifuga* are currently widely available for sale as a dietary supplements used for the treatment of menopause and postmenopausal symptoms. Studies to identify the modes of action and their specific cellular or molecular targets are in progress.

## 3. Experimental

### 3.1. General

Melting points were determined on a PHMK 79/2288 micro-melting point apparatus and uncorrected. Optical rotations were measured with a SEPA-300 polarimeter at room temp. UV spectra were obtained in MeOH with a Shimadzu UV-210 spectrometer and absorption maxima are given in nm. IR spectra were recorded in KBr on a Perkin-Elmer 577 spectrometer. FABMS were run on a VG AUTOSPEC 3000 mass spectrometer. ^1^H and ^13^C NMR spectra were measured on a Bruker AM-500 spectrometer at 500.13 and 125.77 MHz, respectively equipped with an indirect detection probe. Chemical shifts were referenced to the solvent signals. The hetereonuclear HSQC spectra were optimized for an average ^1^*J*_CH_ of 140 Hz; the gradient-enhanced HMBC experiments were optimized for a ^3^*J*_CH_ of 8 Hz.

### 3.2 Plant materials

The plant materials used in for the current research were collected from the prefecture of Dali in Yunnan province. A voucher specimen has been deposited in the herbarium of Kunming Institute of Botany, the Chinese Academy of Sciences. The identity of the plant materials were identified as *C. foetida* by Prof. S.-J. Pei, Kuming Institute of Botany.

### 3.3. Extraction and isolation

The powdered dried roots/rhizomes (2.8 kg) were extracted with MeOH (3 × 10 L) overnight under reflux. Removal of the solvent afforded a black syrup-like residue (459 g) which was then suspended in water-MeOH (9:1, 1.3 L) and partitioned successively using EtOAc (1.8 L × 3) and *n*-BuOH (0.8 L × 3) to afford EtOAc-soluble (214 g) and *n*-BuOH-soluble fractions (82 g). The EtOAc-soluble fraction was absorbed on silica gel (300 g) and subjected to column chromatography eluting with a gradient system of CHCl_3_-MeOH with a increasing percentage of MeOH from zero to 100% to give six fractions (Fr.s. 1–6). Compounds **1** (19 mg) and **2** (23 mg) were obtained as amorphous powders from fraction 3 after repeated chromatography using a combination of silica gel and RP_18_ reverse-phase columns. During the process of separation of compounds **1** and **2**, seven known compounds were also isolated as well.

### 3.3.1. Cimicifoetiside A (1)

White amorphous powder; m. p. 143–145°C; 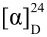 25.0° (*c* = 0.80, MeOH); IR (KBr):ν_max_: 3443, 2963, 2934, 2870, 1739, 1735, 1457, 1239, 1070 cm^-1^; HRFABMS (*m/z*) 619.3854 [M-H-OAc]^-^ (calcd for C_35_H_55_O_9_, 619.3846). ^13^C and ^1^H NMR data were shown in Table 1.

### 3.3.2. Cimicifoetiside B (2)

White amorphous powder; m. p. 157–159°C; 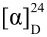 41.1 (*c* = 0.75, MeOH); IR (KBr): ν_max_ = 3444, 2961, 2936, 1739, 1313, 1200, 1166, 604 cm^-1^; HRFABMS (*m/z*) 661.3944 [M-H-OAc]^-^ (calcd for C_37_H_57_O_10_, 661.3951); ^13^C and ^1^H NMR data were shown in Table 1.

## Supporting Information

File 1 Experimental detail of MTT assay. The cytotoxicity of compounds 1, 2 were evaluated using an established protocol with a minor modification.
