# Supplementary material for: Cimicifoetisides A and B, two cytotoxic cycloartane triterpenoid glycosides from the rhizomes of Cimicifuga foetida, inhibit proliferation of cancer cells
Source: Beilstein J Org Chem. 2007 Jan 31;3:3. doi: 10.1186/1860-5397-3-3 (PMC1803790; doi:10.1186/1860-5397-3-3)
Supplement: File 1 — Experimental detail of MTT assay. The cytotoxicity of compounds 1, 2 were evaluated using an established protocol with a minor modification. [file Beilstein_J_Org_Chem-03-03-s001.doc]

Additional file #1

Cimicifoetisides A and B, Two Cytotoxic Cycloartane Triterpenoid Glycosides from the Rhizomes of *Cimicifuga foetida,* Inhibit Proliferation of Cancer Cells

Li-Rong Sun,^1^ Chen Qing,^2^ Yan-Li Zhang,^2^ Shu-Yu Ji,^2^ Zhong-Rong Li,^1^ Shen-Ji Pei,^1^

Ming-Hua Qiu,^1^* Michael L. Gross^3^ and Samuel X. Qiu^3, 4*^

*^1^State Key Laboratory of Phytochemistry and Plant Resources in West China, Kunming Institute of Botany, The Chinese Academy of Sciences, Kunming, Yunnan, P. R. China;*

*^2^Yunnan Pharmacological Laboratory of Natural Products, Kunming Medical College, Kunming, Yunnan, P. R. China;*

^3^*Chemistry Department, Washington University, Campus box 1134, One Brookings Drive, St. Louis, MO 63130, USA.*

*^4^Natural Products Drug Discovery, Herbstandard, Inc.,* 12305 New Avenue, Suite K., Lemont, IL 60439, USA.

**Cancer cell proliferation inhibition assay**: the cytotoxicity of all of the nine compounds was evaluated against the rat tumor EAC cell, and the human SGC7901 and A231 cancer cell lines using the MTT method (Likhitwitayawuid et al., *J. Nat. Prod*. 1993, **56**: 30-38). Briefly, an improved MTT [3-(4, 5-dimethylthiazol-2-yl)-2, 5-diphenyltetrazolium bromide] colorimetric assay was performed in 96-well plates. The assay is established on the reduction of MTT by the mitochondrial dehydrogenase of viable cells to yield a blue formazan product that can be measured spectrophotometrically. In the experiment, the negative reference agents were isochoric normal saline, 1% DMSL, or 0.1% DMSO, and *cis*-platin was used as the positive reference substance with concentrations of 0.1 μg/mL, 1 μg/mL, and 10 μg/mL. EAC, SGC7901 and A231 cells at the log phase of their growth cycle (5 × 10^4^/mL) were added to each well (90 μL/well), then treated in four replicates at various concentrations of the drugs (10^-4^, 10^-5^, 10^-6^, 10^-7^, 10^-8^ mol/L) with six vacant reference wells set in one plate (100 μL cultured media each well), and incubated for 48 h at 37 °C in a humidified atmosphere of 5% CO_2_. After 48h, 10μL of MTT solution (5 mg/mL) were added to each well, which was incubated for another 4 h. Then, a three-system solution of 10% SDS-5% isobutanol-0.012 mL/L HCl (w/v/v) was added to each well (100 μL/well). 12 h later at room temperature, the OD value of each well was recorded on an ELISA reader (Bioteck EL-340, U.S.A.) at two wavelengths (570 and 630 nm).
